# Molecular characterization and phylogenetic analysis of foot-and-mouth disease virus in Nigeria

**DOI:** 10.1186/s12985-026-03226-x

**Published:** 2026-07-03

**Authors:** Adebayo Emmanuel Sopeju, Adewale Temitope Adebare, Oluwaseun Akinmadewa Ala, Babatunde Olanrewaju Motayo, Andrew Raymond Peters, Anyebe Bernard Onoja, Adedayo Omotayo Faneye

**Affiliations:** 1 Department of Virology, Faculty of Basic Medical Sciences, University of Ibadan, Ibadan, Nigeria; 2 Department of Medical Laboratory Science, Chrisland University, Abeokuta, Nigeria; 3 Arpexas (Scotland) Limited, Scotland, UK

**Keywords:** FMD, Serotyping, Recombinant strain, Phylogenetic analysis, Nucleotide sequencing

## Abstract

Foot-and-Mouth Disease (FMD) is a viral disease that affects various cloven-hoofed animals, causing huge economic losses in the livestock sector worldwide. Foot-and-Mouth Disease virus (FMDV), the causative agent of FMD, is recognized to have seven antigenically and immunogenically distinct serotypes, namely, A, O, C, Asia 1, SAT 1, SAT 2, and SAT 3. There are considerable antigenic and genetic diversities within each serotype, resulting in numerous subtypes. This study was aimed at providing updated information on the circulating FMDV strains in Nigeria. We collected 298 epithelial tissue samples aseptically from the oral cavity of cattle suspected of having FMD based on clinical manifestations from December 2023 to November 2024 in representative states of four geopolitical zones in Nigeria. RNA extraction, molecular detection, molecular serotyping, nucleotide sequencing, phylogenetic, and other deep bioinformatic analyses were carried out. Out of the 298 epithelial tissue samples extracted and amplified, a total of 73 (24.5%) samples were positive for FMDV, and 44 (60.3%) were further analyzed. Six out of the 44 samples were sequenced, which, after BLAST analysis, all yielded serotype A, based on VP1 sequences. Phylogenetic analysis placed the study sequences in a distinct cluster separate from the previously reported Nigerian strains, but showed that they evolved from the A/Africa genotype IV topotype. Our analysis also identified a recombinant isolate (PX872163) that shared 97.3% and 93.1% nucleotide identity with PX872165, a major parent, and PX872166, a minor parent, respectively. Five putative B-cell epitopes were identified within the VP1 capsid protein, two of which were localized within the loop regions. In addition, a novel epitope, _162_(RAESIQE)_168_, was identified adjacent to the G-H loop. This study provides the first evidence for the circulation of a recombinant FMDV serotype A in Nigeria and suggests that both genetic recombination events and antibody-mediated immune pressure may be important drivers shaping FMDV evolution in the country.

## Introduction

Foot-Mouth-Disease (FMD) is a viral disease that affects various cloven-hoofed animals and causes huge economic losses in the livestock sector worldwide. It is caused by a virus, Foot-and-Mouth Disease virus (FMDV), from the genus *Aphthovirus* in the family *Picornaviridae*, which has a single-stranded RNA genome of approximately 8.5 kb [7, 16, 17]. The virus is known to be one of the most fearful viral pathogens of animals due to its high contagiousness, near 100% morbidity, close to 50% mortality in young animals [53] among all ungulates (domestic and wild) [4, 7],global distribution and severe economic impacts [3, 51] especially among smallholders farmers, the highest holders of domestic ungulates in Nigeria [55]. FMDV is recognized to have seven antigenically distinct serotypes namely: A, O, C, and Asia 1 (Euroasiatic serotypes), SAT 1, SAT 2, and SAT 3 (South African Territories serotypes). All the seven FMDV serotypes differ in their antigenicity, virulence, and host range [35]. Serotype C has not been detected since 2004 and is now considered to be extinct [66]. Besides, there are considerable antigenic and genetic diversities within each serotype resulting in more numerous subtypes [7]. This reveals the quasispecies dynamic nature of this virus which is due to its high mutation rate estimated to be approximately one base misincorporation per genome replication [21]. The high mutation rate leads to a diverse viral population with the attendant challenge on disease management. Firstly, the resulting diversity means that an animal recovered from infection with one serotype or subtype of the same serotype may become resistant to challenge by the same serotype or subtype but remains susceptible to infection by any other serotypes and subtypes of the same serotype [6]. Secondly, there is reduced vaccine effectiveness [1] and increased potential of immune escape mechanisms [8]. Lastly, it complicates the established diagnostic methods [26, 29, 52] posited that there is need for proper understanding of the epidemiology of any disease to formulate most effective control strategies for the disease, and nucleotide sequencing and phylogenetic analysis are useful for the epidemiological investigations as they are more precise for characterizing FMDV strains. Therefore, the continuous study on the detection of the circulating strains using sequencing and phylogenetic analysis coupled with assessment of the effectiveness of the local FMD vaccine are central in the fight against this mutation-prone virus. The detection of the viral serotype and subtypes must be rapid, sensitive and specific for effective disease control and adequate vaccine selection [47]. This paper is therefore aimed at identifying and characterizing FMDV in Nigeria using nucleotide sequencing and phylogenetic analysis to confirm the currently circulating serotypes and strains of FMDV in Nigeria.

## Methodology

### Clinical specimens

Using a sterile scalpel blade, epithelial tissue samples were aseptically collected from the oral cavity of 298 cattle suspected of having FMD based on clinical manifestations from December 3, 2023, to November 30, 2024, in representative states of four geopolitical zones (North-West, North-Central, North-East, and South-West) in Nigeria. Main clinical signs observed were profuse salivation, oral lesions (oral vesicles, ulceration, or mucosal erosion), and lameness. The samples were transported in either Virus Transport Medium (VTM) or commercially available RNA/DNA shield (Zymo Research®, USA). These samples were used for direct RNA extraction following the procedure described by the manufacturer. Ethical approval was obtained from the Ethical Review Board of the National Veterinary Research Institute (NVRI), Vom, with reference number AEC/02/148/24.

### RNA extraction

RNA was extracted directly from a total of 298 epithelial tissues by using the RNeasy Mini kit (QIAGEN GmbH, Hilden, Germany) following the manufacturer’s instructions. RNA was eluted in an elution buffer, and the extract was stored at –80 °C. 

### Oligonucleotide primers

Different primer sets were used for specific detection and serotyping of FMDV in the extracts. First, a specific FMDV universal primer set was used to verify the presence of the etiological agent in the extracts. The universal primers, namely pan-FMD, were selected to amplify a 328 bp DNA fragment within sequences encoding for the 3D gene from any FMDV, regardless of its serotype [46]. Second, at least one FMDV serotype-specific primer set was chosen according to Knowles and Samuel [28] to amplify VP1 regions of the genome for serotypes O, A, C, SAT 1, SAT 2, SAT 3, and Asia 1. Although, these primers have been reported to cross amplify VP1 sequences from other FMDV serotypes (Knowles et al., [31]). Primer sequences are presented in Table 1.

**Table 1:** Primer sets used for the analysis

Name	Primer set	Expected band size	Position	Reference
Pan-FMD	F—GCCTGGTCTTTCCAGGTCT R—CCAGTCCCCTTCTCAGATC	328 bp	3D 5’ UTR	[46]
First serotype primer set (P1)—For detection and sequencing				
Serotype O	F—ACCAACCTCCTTGATGTGGCT	1301 bp	VP1	Yousef et al., [69]
Serotype A	F—TAGCGCCGGCAAAGACTTTGA	824 bp	VP1	Knowles et al., [30]
Serotype C	F- TACAGGGATGGGTCTGTGTGTACC	883 bp	VP1	Knowles et al., [31]
Serotype Asia 1	F—TACACTGCTTCTGACGTGGC	888 bp	VP1	Valarcher et al., [62]
O, A, C, Asia 1	R—GACATGTCCTCCTGCATCTG		2B	
Serotype Asia 1	F – GACACCACTCAGGACCGCCG F – GACACCACACAAGACCGCCG R—AGCTTGTACCAGGGTTTGGC	296 bp	VP1	[47]; Ularamu et al., [60]
Serotype SAT 1	F—CCACATACTACTTTTGTGACCTGGA R—ACAGCGGCCATGCACGACAG	691 bp	VP1 2B	El-kholy et al., [12]
Serotype SAT 2	F – TGAYCGCAGTACACAYGTYC R—GACATGTCCTCCTGCATCTGGTTGAT	666 bp	1D 2B	Shehata et al., [54]
Serotype SAT 3	F – TTCGGTAGGCTGTTGTGTG F – TTCGGGAGACTGTTGTGCG R—GAAGGGCCCAGGGTTGGACTC	201 bp	VP1	Fernandez et al., [14]
Second Sets of primers used for the analysis (P2)—Detection				
O	F—GCAGCAAAACACATGTCAAACACCTT	1165 bp	VP1	Knowles et al., [31]
A	F—TAGCGCCGGCAAAGACTTTGA	814 bp	VP1	Knowles et al., [31]
C	F- TACAGGGATGGGTCTGTGTGTACC	883 bp	VP1	Knowles et al., [31]
Asia 1	F—GCCGGCAARGAYTTTGAGTTYCG	803 bp	VP1	Knowles et al., [31]
Serotype O, A, C, Asia 1	R—GACATGTCCTCCTGCATCTGGTTGAT	-	2B	
SAT 1	F—GTGTACCAGATCACTGACAC	1043 bp	VP1	Knowles et al., [31]
SAT 2	F—TGGGACACMGGIYTGAACTC	1145 bp	VP1	Knowles et al., [31]
SAT 3	F—CTGTACCAAATYACAGACAC	1034 bp	VP1	Knowles et al., [31]
SAT 1–3	R—ACAGCGGCCATGCACGACAG	-	2B	
Sequencing Primer Sets				
O	F—GAYTACGCSTACACSGCGTC			
A	F—TAGCGCCGGCAAAGACTTTGA			
C	F—TACAGGGATGGGTCTGTGTGTACC			
Asia 1	F – GCCGGCAARGAYTTTGAGTTYCG			
SAT 1	F—GTGTACCAGATCACTGACAC			
SAT 2	F—GGTGCGCCGTTGGGTTGCCA			
SAT 3	F—CTGTACCAAATYACAGACAC			
O,A, C, Asia 1, SAT 1–3	R—GAAGGGCCCAGGGTTGGACTC			
Third Sets of primers (P3) - Detection				
SER_O_F	TTTGAGCTCCGCCTACCA	1165 bp	VP1	Designed*
SER_O_R	AGAAGTTGTTTCTCAGGTGCCA	-		
SER_A_F	TTGACCCCCGCACACAAAC	814 bp	VP1	Designed*
SER_A_R	CGTCCCCTGCCAACTTGA			
SER_Asia1_F	CGCCGGCAAAGATTTTGAGT	803 bp	VP1	Designed*
SER_Asia1_R	AGGTCGAAGTTCAGCACCTG	-		
SER_SAT1_F	AGAAGTGGTCACGGTCGATG	1043 bp	2B	Designed*
SER_SAT1_R	CACGTCTCCGGCCAACTTTA	-	VP1	
SER_SAT2_F	ATTCCGGTTCCCTATCGACC	+		Designed*
SER_SAT2_R	CAACGTCTCCTGCCAGTTTCA	-		

^*^The primer sets were designed for this study using free version of Geneious ( https://www.geneious.com/) and NCBI primer design ( https://www.ncbi.nlm.nih.gov/tools/primer-blast/) tools

### Molecular detection of FMDV

Extracted samples were screened for the presence of FMDV genomic RNA using a one-step reverse transcription PCR (RT-PCR) assay. A pan-FMDV primer set (Table 1), targeting the highly conserved 5′ untranslated region (5′UTR) of the viral genome, was employed to enable broad detection across all serotypes. The one-step RT-PCR reaction was carried out in a final volume of 25 µl, comprising 12.5 µl of 2 × qScript XLT One-Step RT-qPCR ToughMix (QuantaBio, USA), 1 µl (10 pmol) of each primer, 5 µl of RNA extract, and 5.5 µl of Rnase-free water. Amplification was performed using a Veriti Pro® Thermal Cycler (Applied Biosystems, USA). Each reaction run included a negative control in which 5 µl of PCR-grade water was substituted for the RNA template. The thermal cycling conditions included an initial reverse transcription step at 50 °C for 30 min, followed by initial denaturation at 95 °C for 15 min. This was followed by 45 cycles of denaturation at 94 °C for 1 min and annealing at 55 °C for 30 s, with an initial extension at 72 °C for 2 min and a final extension at 72 °C for 5 min. Amplified PCR products were subjected to electrophoresis on 1% agarose gel stained with SYBR® Safe (Invitrogen, Thermo Fisher Scientific, USA) and visualized with an Omega Fluor Plus® transilluminator (San Francisco, USA). Samples that yielded the expected 328 bp amplicon band size were subjected to FMDV serotyping.

### Molecular genotyping of FMDV

The FMDV genotyping was achieved through reverse transcription and amplification with the use of the QIAGEN OneStep RT-PCR kit (Qiagen, Hilden, Germany). An amount of 2 μl of extracted RNA was added to the RT-PCR mixture consisting of 1 µl of OneStep RT-PCR Enzyme Mix, 5 µl of OneStep RT-PCR buffer, and 1 µl of 10 mM dNTP mix. The volume was completed to 25 µl with nuclease-free water. The thermal cycling conditions included an initial reverse transcription step at 50 °C for 30 min, followed by initial denaturation at 15 min for 95 °C. This is followed by 45 cycles of 94 °C for 1 min, 60 °C for 1 min (for serotype O) or 55 °C for 1 min (for serotypes A, C and Asia 1) or 50 °C for 1 min (for SAT 1, 2, and 3), and then 72 °C for 2 min. A final extension step was performed at 72 °C for 7 min. The resulting amplicons were visualized following agarose-gel electrophoresis as described earlier.

### Direct sequencing of RT-PCR amplicons

Amplified PCR products were purified using the ExoSAP-IT™ PCR Product Cleanup Reagent (Applied Biosystems, Thermo Scientific, USA), which was followed by Cycle Sequencing using Applied Biosystems BigDye™ Terminator v3.1 Cycle Sequencing Kit. Samples were sequenced in both forward and reverse directions with the sequencing primers listed in Table 1. Post-Cycle Sequencing Purification was done using the Ethanol/EDTA/Sodium Acetate Precipitation method and resuspended in HI-Di Formamide. Capillary electrophoresis was performed in a 3500xL Genetic Analyzer (Applied Biosystems, Foster City, California). The raw sequences were further trimmed and aligned in BioEdit software version 7.2.5.1 [18]. Pairwise percentage identity among the sequenced samples was also calculated in BioEdit. The cleaned sequences generated in this study were subsequently submitted to GenBank under the accession numbers PX872161-PX872166.

### Analysis of selection pressure sites in the VP1 protein codon

Episodic diversifying selection at individual codon sites was assessed using the Mixed Effects Model of Evolution (MEME) implemented in HyPhy via the Datamonkey web server (http://www.datamonkey.org). In addition to MEME, Fixed Effects Likelihood (FEL) and Single Likelihood Ancestor Counting (SLAC) methods were used to evaluate whether individual codons were evolving under purifying (negative) or diversifying (positive) selection. Selection pressure was evaluated based on the dN/dS ratio, which compares the rates of nonsynonymous (dN) and synonymous (dS) substitutions. Of the six VP1 encoding FMDV sequences submitted to the datamonkey server, two sequences with 100% homology were automatically removed, leaving four VP1 sequences for selection analysis. Codon sites with a dN/dS ratio > 1 were inferred to be under positive (diversifying) selection, those with a dN/dS ratio < 1 were interpreted as being under negative (purifying) selection, while sites with a dN/dS ratio equal to 1 were considered to be evolving under neutral evolution. Sites with p-values ≤ 0.1 were inferred to be under statistically significant positive selection. To minimize the likelihood of false‐positive signals in our analysis, statistical significance was assessed using 500 site‐level parametric bootstrap replicates. The best-fit nucleotide substitution model was inferred by the server based on the internal model selection function and the Akaike information criterion [63].

### Detection of recombination

The complete coding region of the VP1 sequence alignment was screened for any evidence of recombination using the RDP5 software v.5.30 [38]. The analysis was performed using various methods available and their default parameters. A recombination event was considered credible only if it is supported by at least three of the seven available methods (RDP, GENECONV, BootScan, MaxChi, Chimaera, SiScan, and 3Seq) with statistically significant p-values (p ≤ 0.05) following Bonferroni correction. The use of all the available methods increases confidence and robustness of the recombination analysis. The RDP, MaxChi, and Chimaera employ a sliding window approach to scan for recombination in polymorphic sites for every potential triplet of a sequence. GENECONV detects potential gene conversion events by identifying unusually long regions of sequence similarity between aligned sequence pairs. The 3SEQ method uses a non-parametric framework to identify significant recombination breakpoints based on ranked clustering statistics. Bootscan projects conflicting phylogenetic signals in different regions of an alignment, while SiSscan identifies shifts in phylogenetic signals that may arise from recombination events, indicating the complexity of evolutionary relationships among the sequences being analyzed.

### Estimation of sequence variability index

The VP1 amino acid sequences of FMDV serotype A obtained in this study were aligned using the BioEdit ClustalW algorithm [18]. Protein sequence variability was assessed using the Protein Variability Server (PVS) based on the Wu–Kabat variability coefficient [15]. This index was calculated using the formula: Variability = N × k/n, where N represents the total number of sequences in the alignment, k denotes the number of different amino acids observed at a specific position, and n corresponds to the frequency of the most common amino acid at that position [25]. The resulting variability scores were used to determine whether the predicted epitopes were positioned in the least variable, moderately variable, or hypervariable regions. In addition, a consensus sequence was generated from the alignment using the PVS server. This consensus sequence was constructed by selecting the most frequently occurring amino acid residue at each position in the alignment and was subsequently used for the 3D structural modeling of the VP1 protein.

### Homology modeling and structural evaluation of VP1 protein

The consensus sequence generated by the Protein Variability Server (PVS) was used in the construction of the FMDV serotype A VP1 three-dimensional (3D) structure on the SWISS-MODEL web server [64]. The quality of the 3D construct was validated with the Ramachandran plot within the VADAR server version 1.8 [65] to evaluate the stereochemical reliability of the modeled structure.

### Detection of B-cell epitopes

Several computational tools and algorithms have been developed for the identification of antigenic fragments within protein sequences. Conformational B-cell epitope prediction in this study was performed using ElliPro within the immune epitope database [43] with default parameters. ElliPro is a web tool that utilizes Thornton’s method and, together with a residue clustering algorithm, the MODELLER program, and the Jmol viewer, allows the prediction and visualization of antibody epitopes in a given protein sequence or structure. ElliPro has been tested on a benchmark dataset of discontinuous epitopes inferred from 3D structures of antibody-protein complexes.

### Evaluation of antigenic diversity

The spatial positions of the predicted B-cell epitopes were examined within the 3D structure of the VP1 protein. The predicted epitopes were assessed for the presence of amino acid substitutions among the analyzed isolates and for potential combinations of mutations occurring within the same epitope. To further characterize sequence diversity at these sites, the average Wu–Kabat protein variability index was calculated for each predicted epitope position [25].

### Phylogenetic analysis

Phylogenetic analysis of the 1D gene (VP1 encoding) was performed in MEGA version 12.1 [33] to assess the evolutionary relationships between the sequences obtained in this study and the reference strains previously reported. A curated dataset comprising 80 VP1 gene sequences retrieved from GenBank, along with six novel sequences generated in this study, yielded a total of 86 sequences for analysis. Multiple sequence alignment was conducted using the Clustal algorithm with default settings. The optimal substitution model for the aligned sequences was determined using the MEGA internal model selection tool, which evaluated key criteria including the Bayesian Information Criterion (BIC) and Akaike Information Criterion (AIC). The General Time Reversible model with gamma-distributed rates and invariant sites (GTR + G + I) was selected as the best fit model, as it showed the lowest BIC score. A maximum likelihood (ML) phylogenetic tree was then inferred under this model with branch support evaluated using 1,000 bootstrap replicates. The tree was subsequently visualized and annotated in the ITOL v7 program.

## Result

Out of the 298 epithelial tissue samples extracted and amplified using the one-step PCR technique, a total of 73 samples were positive, out of which 44 (60.3%) were further analyzed. Six out of the 44 samples were sequenced, which, after BLAST analysis, all yielded serotype A, based on VP1 sequences.

### Genotyping

Using specific primer sets as indicated above (Table 1), we carried out genotyping (for serotypes O, A, C, SAT 1, SAT 2, SAT 3, and Asia 1) on all the 44 FMD-positive samples. Out of the three primer sets used for each serotype, the second primer set (P2) gave the highest band size that passed at the sequencing level. (Table 2). In all, P1 generated 27 quality bands, but only one passed at the sequencing level; P2 generated 14 quality bands, and six passed at sequencing, while P3 did not give any quality bands that could be taken forward to sequencing. All the six samples that passed at sequencing level gave similar sequences, which were for serotype A.

**Table 2:** Serotyping result based on band size and primer sets

Serotype	Primer sets	Total	Pos (N^)	Pos (%)	No passed
O	P1	44	23 (6)	52.3%	0
	P2	23	2 (2)	8.7%	0
	P3	10	0	0.0%	-
A	P1	44	15 (5)	34.1%	0
	P2	18	8 (5)	44.4%	1
	P3	4	0	0.0%	-
C	P1	44	1 (1)	2.3%	0
	P2	13	1 (1)	7.7%	1
	P3	0	0		-
Asia 1	P1	44	31 (10)	70.5%	1
	P2	15	4 (4)	26.7%	3
	P3	10	0	0.0%	-
SAT 1	P1	44	1 (0)	2.3%	-
	P2	18	2 (1)	11.1%	0
	P3	6	0	0.0%	-
SAT 2	P1	44	2 (2)	4.5%	0
	P2	24	1 (1)	4.2%	0
	P3	7	0	0.0%	-
SAT 3	P1	44	8 (3)	18.2%	0
	P2	19	1 (1)	5.3%	0
	P3	0	0		-

^Number of quality band results taken forward to sequencing; “No Passed” represents the number of quality bands that passed sequencing

### Protein sequence analysis

**Fig. 1:**
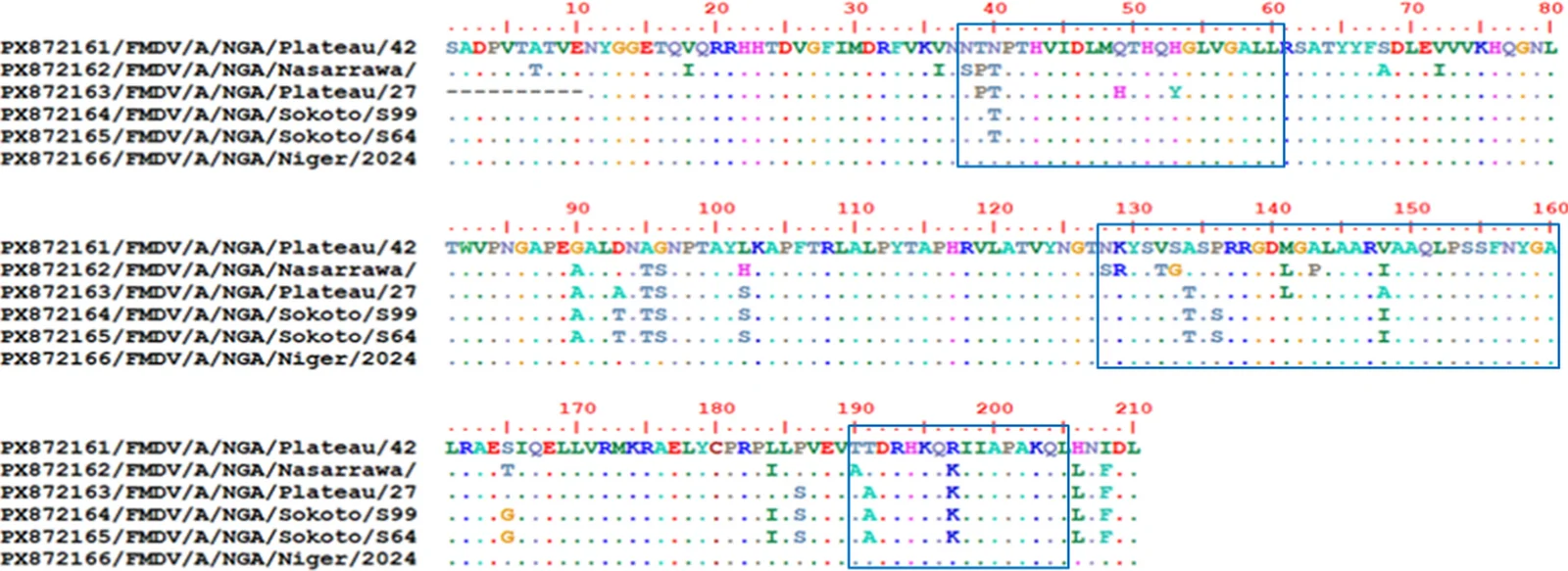
Deduced amino acid sequence alignment of the VP1 protein of the novel FMDV serotype A strains identified in this study. The difference at each amino acid position between the sequences are shown in single letter code. Conserved residues are shown as dots, deletions are indicated as dashes, while the neutralizing antigenic sites across the aligned sequences are highlighted with blue boxes

**Fig. 2:**
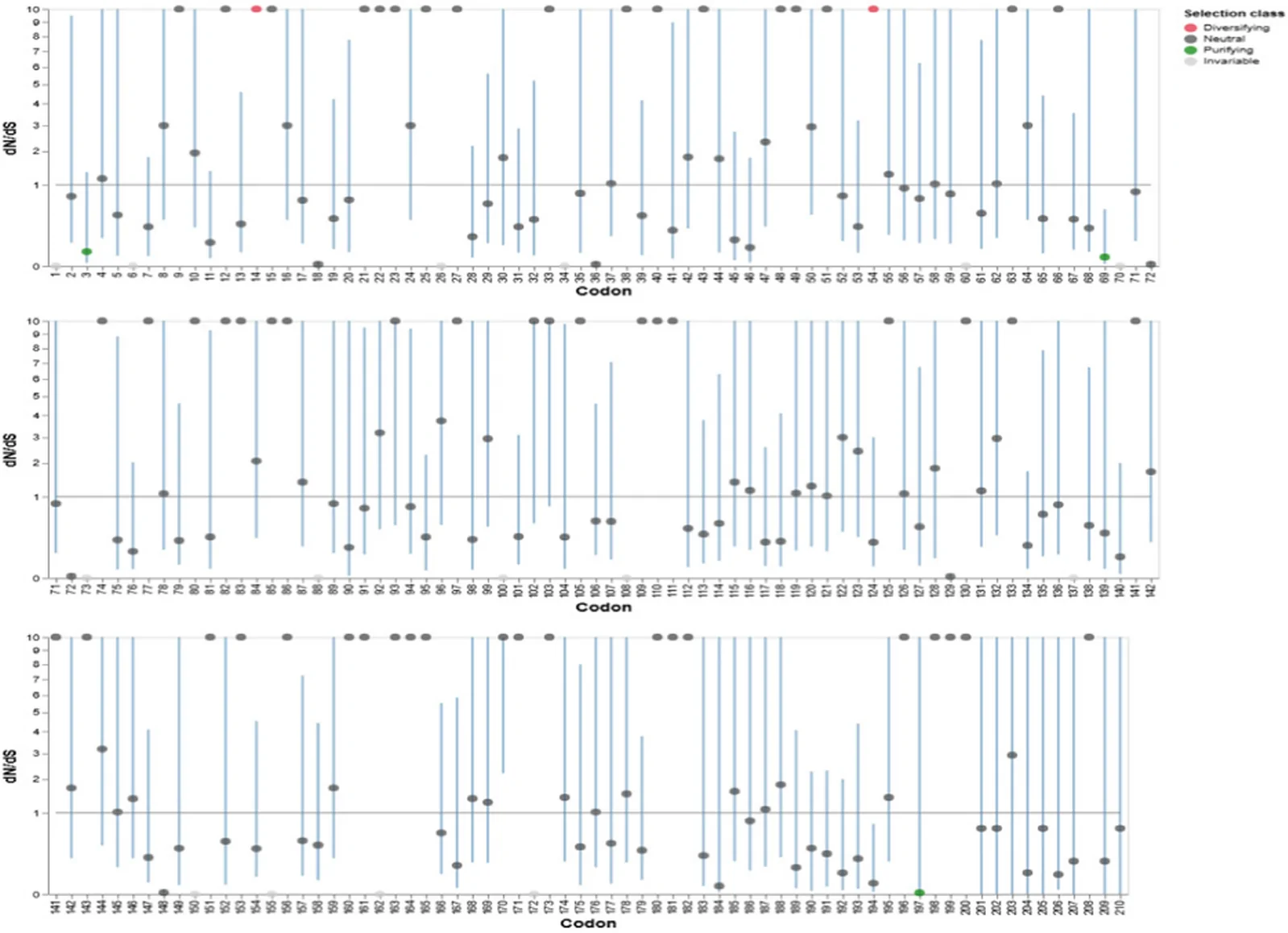
Fixed effect likelihood (FEL) estimates of dN/dS at each codon site, together with estimated profile confidence intervals. dN/dS = 1 (neutrality) is depicted as a horizontal gray line. Boundaries between partitions are shown as vertical dashed lines. Positively selected sites are shown as red spheres, while negatively selected sites are shown as green spheres

Mutation analysis of the deduced VP1 amino acid sequences generated in this study showed variations within the major neutralizing antigenic sites of FMDV. These sites include the B-C loop (residues 40–60), G-H loop (residues 130–160), and the C-terminus region (residues 190–210) (Fig. 2). Most of the amino acid substitutions observed within these regions were conserved across the six study sequences, indicating a high level of sequence similarity. Within the B-C loop, non-conservative substitutions were identified only in PX872163 (A/NGA/Plateau/27) at positions Q49H and H53Y. Greater variability was observed within the hypervariable G-H loop. PX872162 (A/NGA/Nasarawa) showed substitutions at positions V132T, S133G, and A143P, while PX872163 (A/NGA/Plateau/27), PX872164 (A/NGA/Sokoto/S99), and PX872165 (A/NGA/Sokoto/S64) shared substitutions at position A134T and P136S. The C-terminus appears to be largely conserved, with only minor mutations observed at positions T190A and H196L. The arginine-glycine-aspartic acid (RGD) motif which is essential for viral attachment to host integrin receptors, was fully conserved across the six sequences (Fig. 1). However, the two leucine (L) residues located at positions + 1 and + 4 downstream of the RGD motif, which play a key role in cell recognition, showed conservative substitution, with the first leucine residue being replaced with methionine (M). In addition, four of the six serotype A isolates (PX872161, PX872164, PX872165, and PX872166) had RGDMGAL, while PX872162 and PX872163 had RGDLGPL and RGDLGAL, respectively.

### Selection pressure analysis

**Fig. 3:**
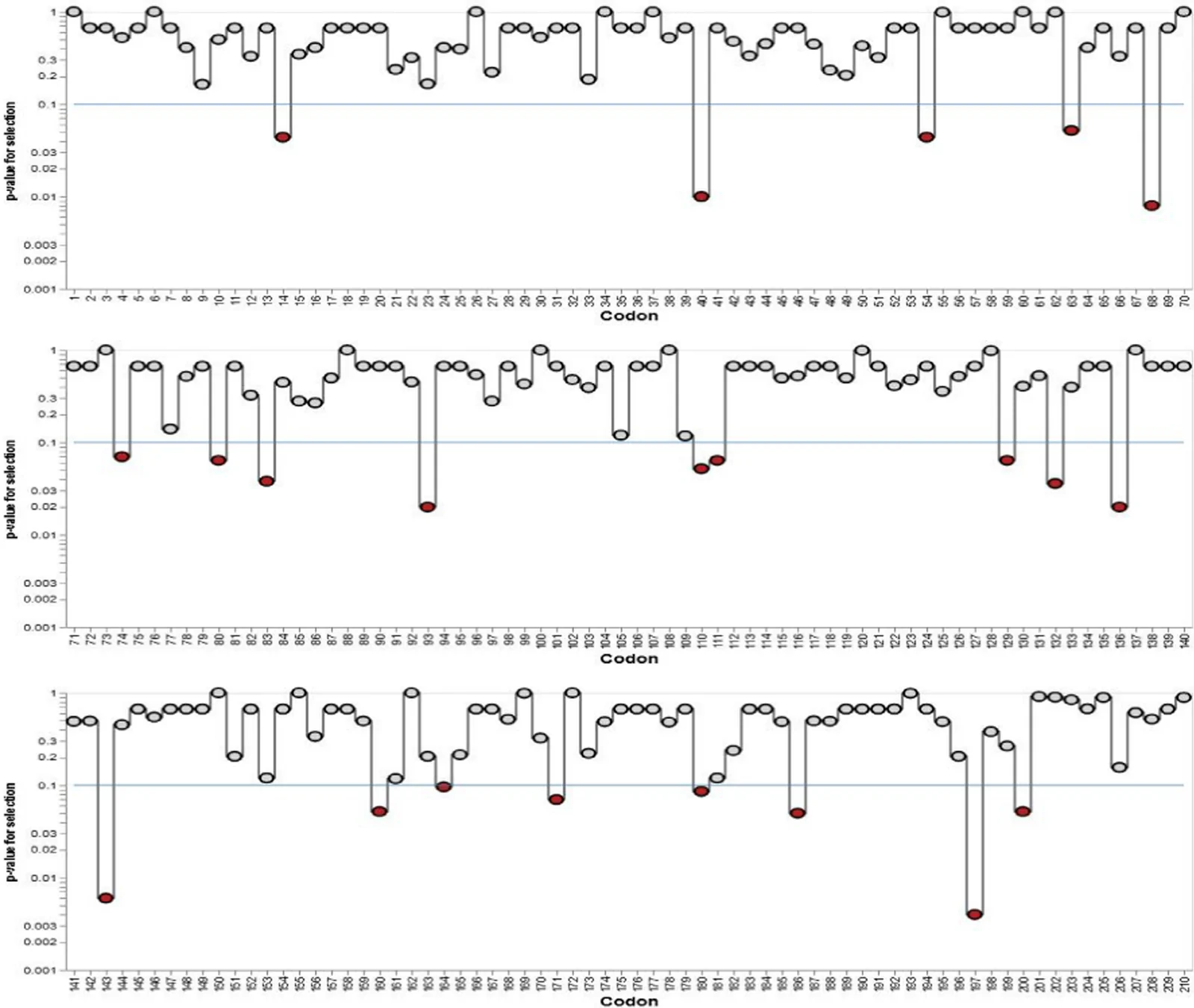
Mixed effect model of evolution (MEME) plot showing codon sites under positive selection. Red spheres indicate positively selected sites. Horizontal blue line marks the significance threshold (p = 0.1). Sites were identified using likelihood-based MEME approach

Site-specific selection pressure acting on the VP1 protein coding gene was assessed using SLAC, FEL, and MEME methods implemented in Datamonkey web server. The SLAC analysis detected zero codon sites under positive selection but identified a single site (position 210) to be under negative selection (data not shown). At a significance threshold of 0.1, the FEL method inferred two codon sites (14 and 54) under positive selection and three additional codon sites (3, 69, and 197) under negative selection (Fig. 2). SLAC and FEL are inherently conservative methods, and as a result, the true false-positive rate is likely to be substantially lower than the nominal significance threshold applied in the analysis [32]. In contrast, the MEME method, which detects episodic diversifying selection at individual codon sites, identified 22 codon positions (14, 40, 54, 63, 68, 74, 80, 83, 93, 110, 111, 129, 132, 136, 143, 160, 164, 171, 180, 186, 197, and 200) under positive selection at a significance threshold of *p* ≤ 0.1 (Fig. 3). No evidence of pervasive positive selection was detected by the three methods used for this analysis.

### Recombination analysis

**Fig. 4:**
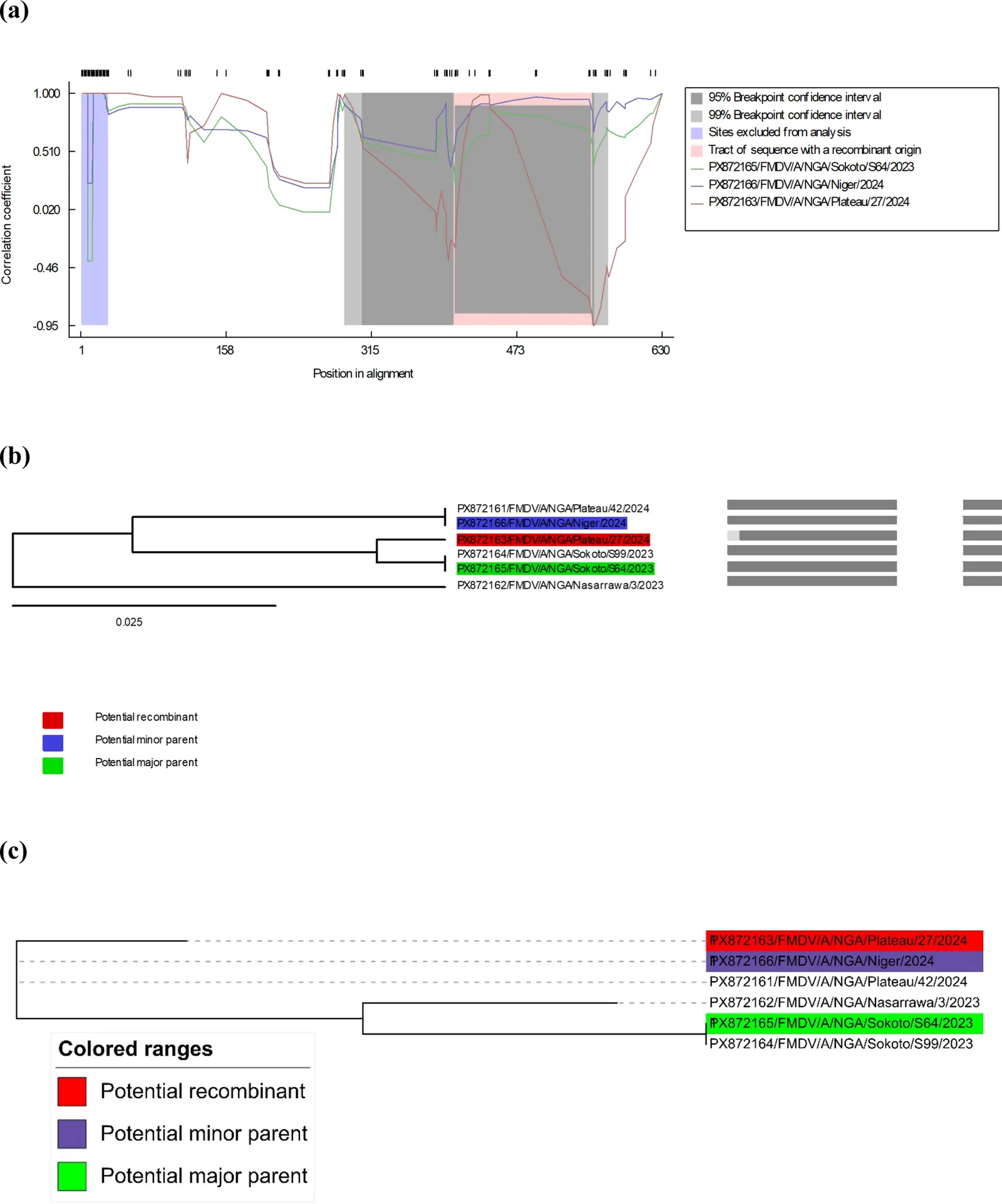
Recombinant analysis of the A/NGA/Plateau/27 isolate based on the complete VP1 gene sequence using the RDP5 software. In (**a**), A/NGA/Plateau/27 serves as the query sequence, while the lines show different pairs of analysis for recombination: green (A/NGA/Sokoto/64), blue (A/NGA/Niger), and coral (A/NGA/Plateau/27). In (**b**), the recombinant tree is presented, with the red area representing A/NGA/Plateau/27 as the recombinant, the green area indicating the A/NGA/Sokoto/64 isolate as the major parent, and the blue area showing A/NGA/Niger as the minor parent. Dark grey areas represent the considered nucleotide site. Light grey areas represent the ignored nucleotide site. White areas highlight breakpoints, indicating specific points where recombination has occurred. The phylogram represents recombination events attributed to the major putative parent in regions 1–403 and 555–630, and minor putative parent region 404–554. In (**c**), the recombinant tree is presented, with the A/NGA/Sokoto/S64 isolate in green, representing the major potential parent and the A/NGA/Niger isolate, in blue as the minor potential parent, with the A/NGA/Plateau/27, in red, representing the potential recombinant strain

Recombination analysis of the complete VP1 coding region revealed evidence of intraserotypic recombination signal within the study sequence. Two putative recombination events were identified in the analysis. However, one event could not be confirmed as the putative parent and recombinant sequences did not overlap in time and space. In the one confirmed recombinant event, the recombinant isolate PX872163 (A/NGA/Plateau/27) had PX872165 (A/NGA/Sokoto/64) as major parent and PX872166 (A/NGA/Niger) as minor parent. The putative major and minor parental sequences shared 97.3% and 93.1% nucleotide identity with the recombinant isolate. The analysis also showed nucleotide recombination at the starting and ending breakpoints in the VP1 sequence at nucleotide positions 404 and 554, respectively, with a 99% CI (Fig. 4a). To further analyze the recombination signal detected by RDP5, separate phylogenetic analyses were performed using genomic regions defined by the identified recombination breakpoints. In the non-recombinant regions (Fig. 4b) (nucleotide position 1–403 and 555–630), the recombinant isolate clustered with the putative major parent, whereas in the recombinant region (Fig. 4c) (nucleotide position 404–554), it clustered with the putative minor parent.

### Estimation of protein variability index

**Fig. 5:**
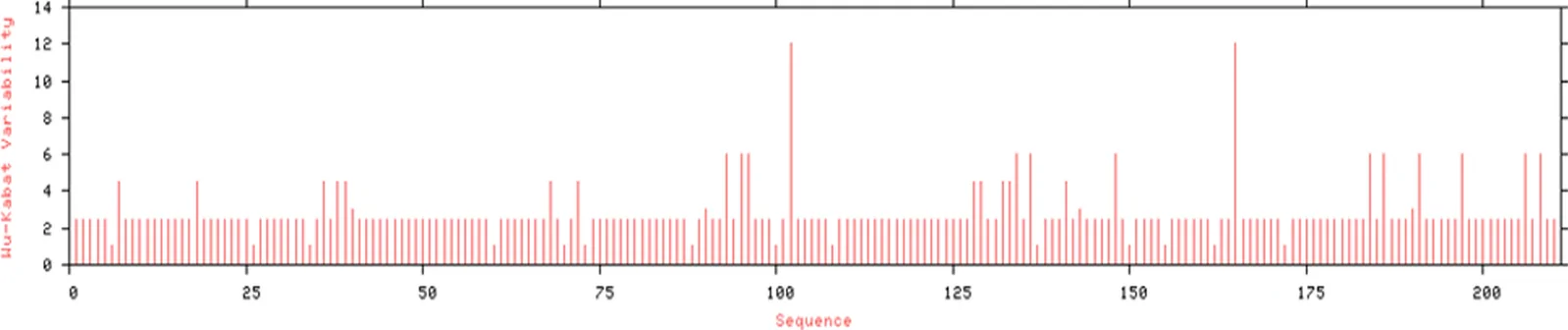
Wu–Kabat protein variability index for each of the 210 amino acid residues of VP1 protein of the FMDV serotype A

Clustal alignment of the FMDV serotype A VP1 sequences generated in this study was uploaded to the Protein Variability Server to calculate the Wu–Kabat variability index. Based on this analysis, a protein variability plot (Fig. 5) was generated using the Wu–Kabat coefficient to assess the distribution of sequence variability across the VP1 protein. The consensus sequence (SADPVTATVENYGGETQVQRRHHTDVGFIMDRFVKVNNTTPTHVIDLMQTHQHGLVGALLRSATYYFSDLEVVVKHQGNLTWVPNGAPEAALDNTSNPTAYSKAPFTRLALPYTAPHRVLATVYNGTNKYSVSASPRRGDMGALAARIAAQLPSSFNYGALRAESIQELLVRMKRAELYCPRPILPVEVTTDRHKQKIIAPAKQLLNFDL) of the VP1 protein was derived from the multiple sequence alignment using the PVS server.

### Homology modeling and structural evaluation of VP1 protein

**Fig. 6:**
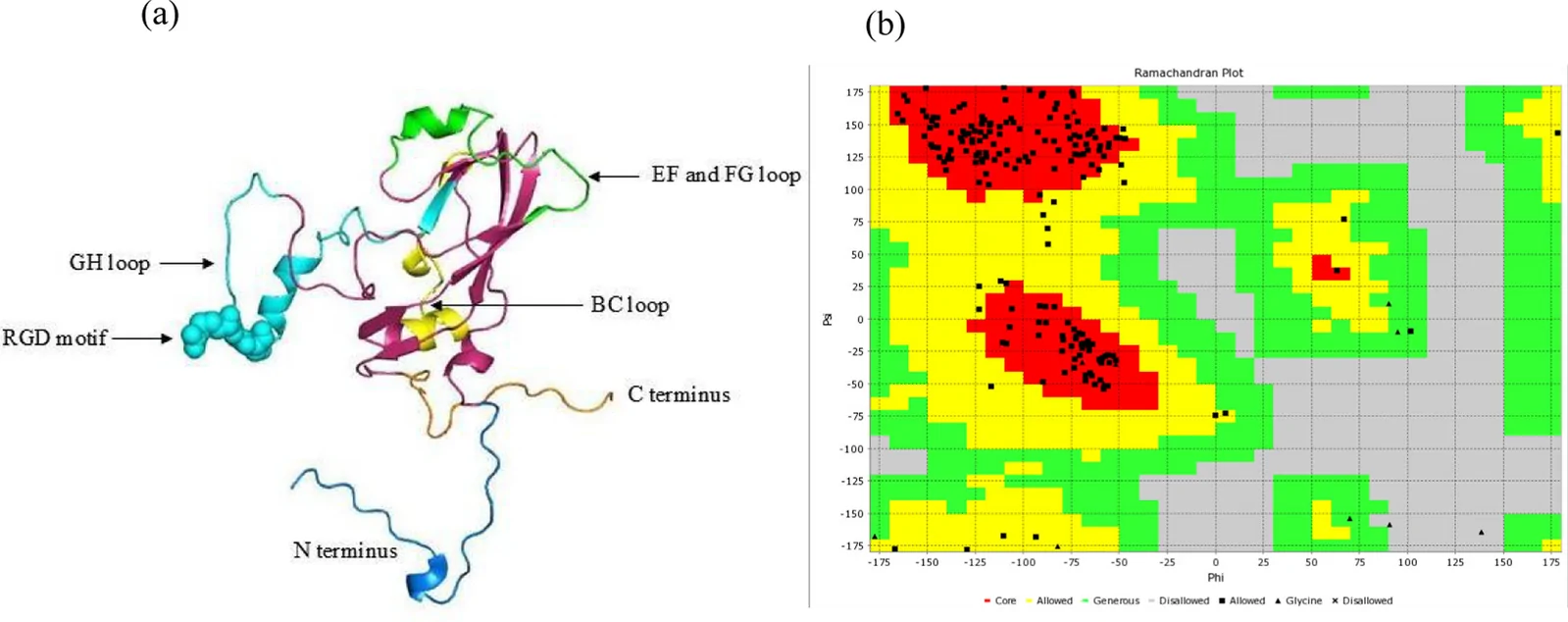
(**a**) Three-dimensional (3D) structure of the FMDV serotype A VP1 consensus sequence. The 3D structure was modeled on the SWISS-MODEL web server and visualized in PyMOL software. (**b**) Ramachandran plot showing the stereochemical properties of the modeled VP1 protein. The plot was generated by the VADAR server

To model the FMDV serotype A VP1 capsid protein, the consensus sequence obtained from the PVS server was uploaded onto the SWISS-MODEL web server. The modeling workflow consists of several steps, including target template identification and sequence alignment, model construction, and model evaluation. As no experimentally determined 3D structure of the VP1 protein of FMDV serotype A was available in the Protein Data Bank, the PDB ID: 1fod (2.60 Å) was used as a target template for the model construction. The template was selected based on its superior coverage (98%) of the target sequence. In addition, this template exhibited 70% sequence identity and 51% sequence similarity with the target sequence, which were higher than those observed for other available templates in the PDB database. The quality of the modeled VP1 3D structure (Fig. 6a) was subsequently evaluated with a Ramachandran plot generated by the VADAR server. The obtained Ramachandran plot (Fig. 6b) statistics demonstrated that 90.3% of amino acid residues are distributed in the most favoured region (Core), 9.1% in the additionally allowed region, 0.6% in the generously allowed region, and 0.0% in the disallowed region.

### Structural mapping of B-cell epitopes

**Fig. 7:**
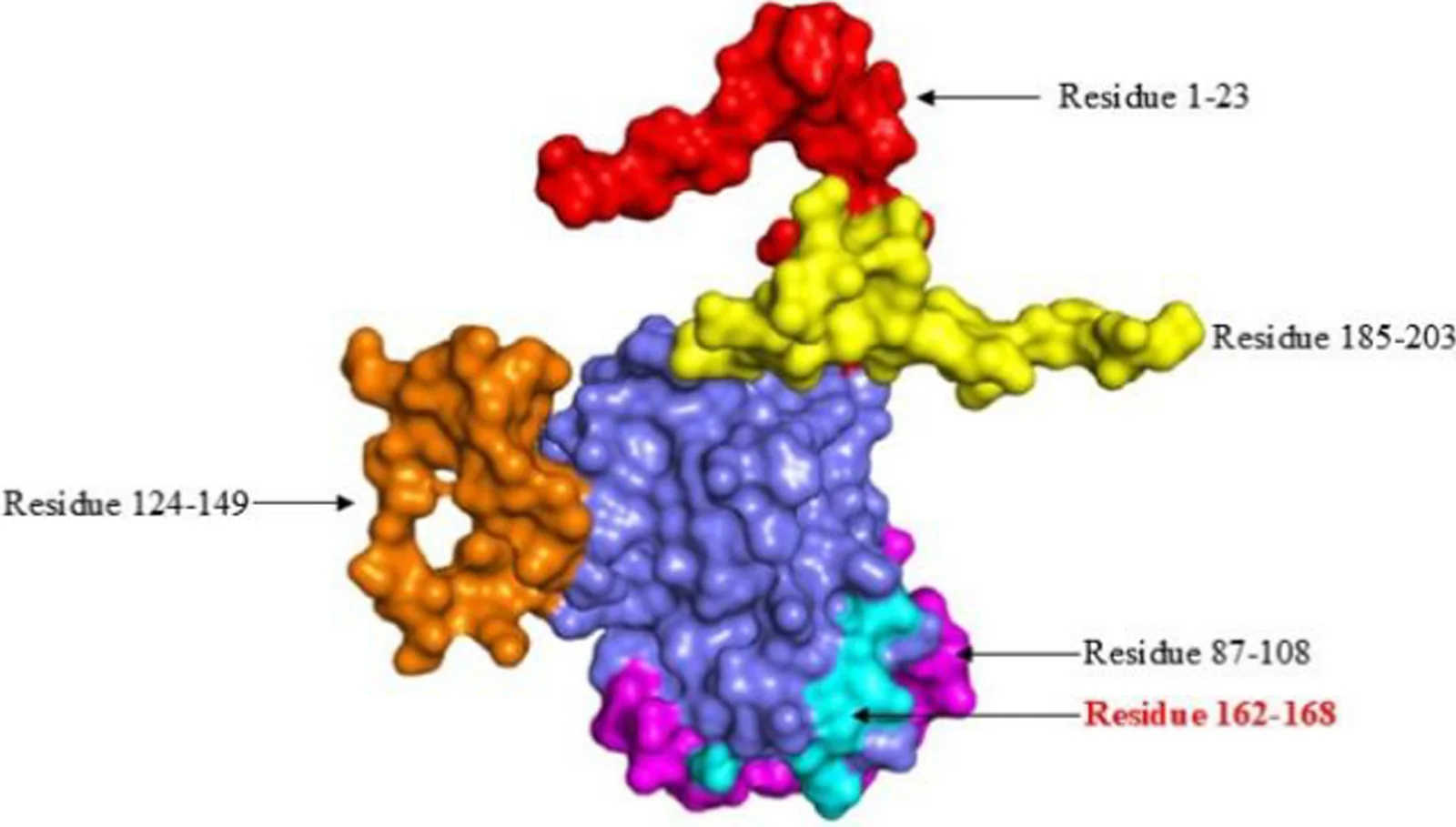
Predicted B-cell epitopes mapped onto the VP1 protein’s three-dimensional (3D) structure, using the PyMOL visualization software

Conformational B-cell epitopes predictions were performed using the ElliPro web server based on the three-dimensional model of the VP1 capsid protein. This analysis identified five putative B-cell epitopes (Table 3). These included one epitope located at the N-terminus, one positioned between the EF and FG loops, one within the GH loop, another adjacent to the GH loop, and one at the C-terminus of the protein. The predicted epitopes were subsequently mapped onto the VP1 3D structure using PyMOL software (Fig. 7).

**Table 3:** Predicted B-cell epitopes showing prediction scores, residue numbers, and location within the three-dimensional (3D) structure

Predicted epitope	Position	Location in loop	Number of residues	ElliPro score
SADPVTATVENYGGETQVQRRHH	1–23	N terminus	23	0.838
APEAALDNTSNPTAYSKAPFTR	87–108	EF and FG loop	22	0.642
YNGTNKYSVSASPRRGDMGALAARIA	124–149	GH loop	26	0.729
RAESIQE	162–168	Post GH loop	7	0.574
LPVEVTTDRHKQKIIAPAK	185–203	C terminus	19	0.802

### Sequence variability analysis

The average Wu–Kabat variability index was estimated for each of the predicted B-cell epitopes within the VP1 protein. Mutation types were also identified, and potential combinations of amino acid substitutions were analyzed based on the Clustal alignment of the VP1 protein sequences (Table 4). The analysis revealed that the epitopes spanning residues 87–108 (APEAALDNTSNPTAYSKAPFTR) and 124–149 (YNGTNKYSVSASPRRGDMGALAARIA) were the most hypervariable of the five predicted B-cell epitopes, with average variability indices of 3.16 and 3.18, respectively. Each of these antigenic peptides exhibited five distinct mutation combinations across the analyzed sequences. In contrast, fewer mutation combinations were observed at the N- and C-terminal regions. The epitope located at the N-terminus (residues 1–23; SADPVTATVENYGGETQVQRRHH) showed only two mutation combinations, whereas the epitope spanning the C-terminus (residues 185–203; LPVEVTTDRHKQKIIAPAK) showed four mutation combinations. Although the epitope adjacent to the G–H loop (residues 162–168; RAESIQE) of the VP1 protein exhibited a relatively high variability index of 3.57, only three mutation combinations were observed within this region.

**Table 4:** Average Wu–Kabat protein variability index, mutation types, and mutation combinations for the predicted B-cell epitopes

Peptide	Average variability index	Mutation type	Mutation combination
_1_(SADPVTATVENYGGETQVQRRHH)_23_	2.52	A7T, V18I	Two combinations: _1_(SADPVTATVENYGGETQVQRRHH)_23_ _1_(SADPVTTTVENYGGETQIQRRHH)_23_
_87_(APEAALDNTSNPTAYSKAPFTR)_108_	3.16 (Hypervariable)	A90G, D93A, D93T, T95A S96G, S102L, S102H,	Five combinations: _87_(APEAALDNTSNPTAYSKAPFTR)_108_ _87_(APEGALDNTSNPTAYHKAPFTR)_108_ _87_(APEGALDNAGNPTAYLKAPFTR)_108_ _87_(APEAALTNTSNPTAYSKAPFTR)_108_ _87_(APEAALANTSNPTAYSKAPFTR)_108_
_124_(YNGTNKYSVSASPRRGDMGALAARIA)_149_	3.18 (Hypervariable)	N128S, K129R, V132T, S133G, A134T, P136S M141L, A143P, I148V, I148A,	Five combinations: _124_(YNGTNKYSVSASPRRGDMGALAARIA)_149_ _124_(YNGTSRYSTGASPRRGDLGPLAARVA)_149_ _124_(YNGTNKYSVSTSPRRGDMGALAARAA)_149_ _124_(YNGTNKYSVSTSSRRGDMGALAARIA)_149_ _124_(YNGTNKYSVSASPRRGDMGALAARVA)_149_
_162_(RAESIQE)_168_	3.57	S165T, S165G	Three combinations: _162_(RAESIQE)_168_ _162_(RAETIQE)_168_ _162_(RAEGIQE)_168_
_185_(LPVEVTTDRHKQKIIAPAK)_203_	3.00	P186S, T190A, T191A, K197R	Four combinations: _185_(LPVEVTTDRHKQKIIAPAK)_203_ _185_(LPVEVTTDRHKQRIIAPAK)_203_ _185_(LPVEVATDRHKQKIIAPAK)_203_ _185_(LSVEVTADRHKQKIIAPAK)_203_

^#^Underlined amino acid letter codes represent the position where substitutions have occurred

### Phylogenetic analysis

The phylogenetic analysis of the FMDV serotype A showed that the isolates obtained in this study do not cluster with the previously reported Nigerian strains, but instead grouped together on a distinct branch that evolved from the A/AFRICA/G-IV topotype. This branching pattern indicates that the study sequences originated from the Nigerian genotype IV lineage but have diverged to form a separate clade. In contrast, the SAT 1 sequences formed an independent cluster, clearly separated from all serotype A sequences, including those generated in this study (Fig. 8).

**Fig. 9:**
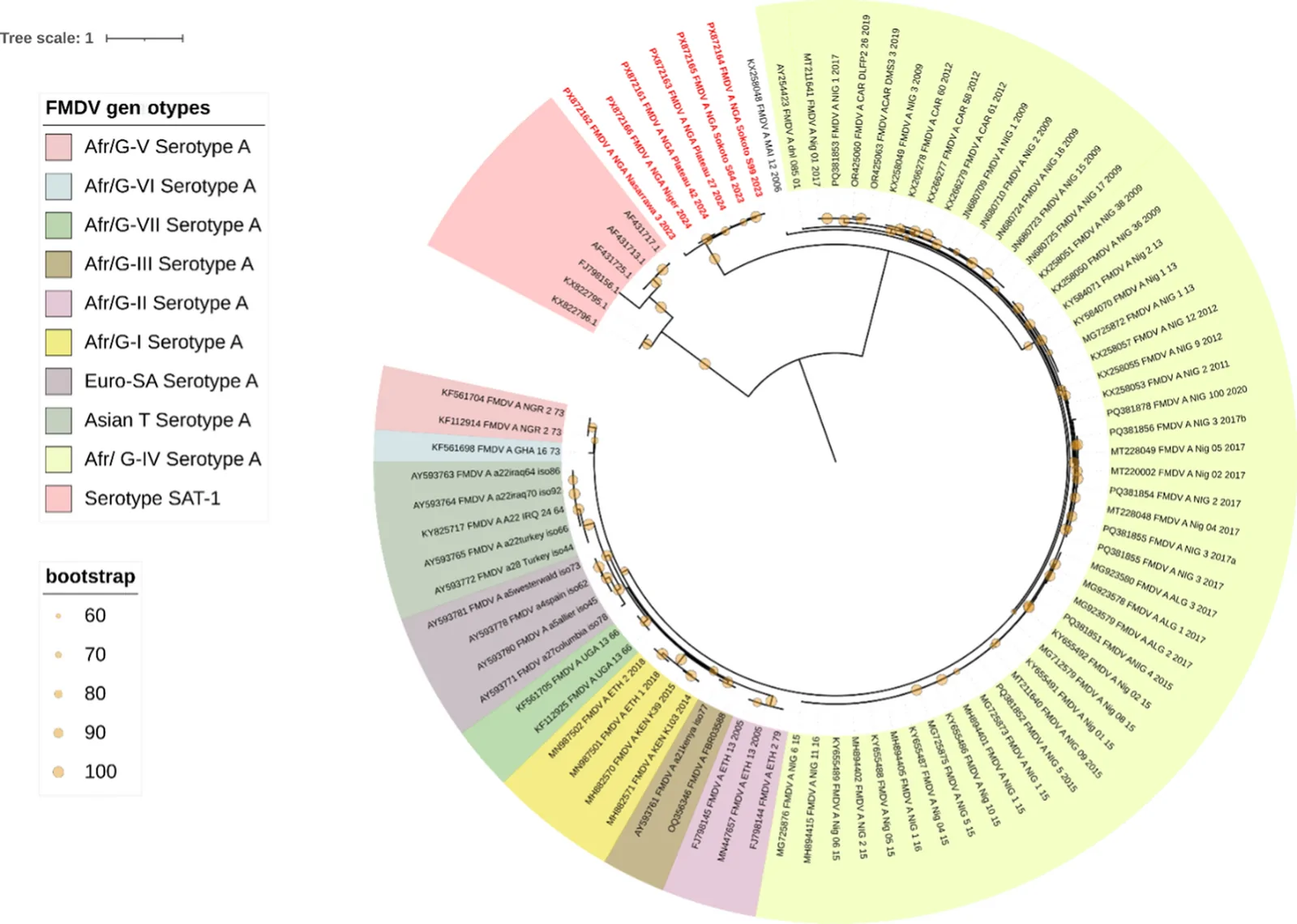
Phylogram of the VP1 gene of FMDV serotype A inferred with maximum likelihood method under the GTR + G + I substitution model. Brown circles at nodes indicate the bootstrap values based on the maximum likelihood analysis of 1000 replicates. Novel sequences from this study are shown in red labels

## Discussion

This study recorded all known serotypes using specific primer sets and their band sizes which was primarily due to the potential cross amplification of VP1 by these primers (Knowles et al., [31]). However, six samples yielded quality sequences, which, after BLASTing, were identified as serotype A with high percentage identity (92.8–100%) and subjected to further analysis. Phylogenetic analysis of the VP1 gene showed a clear genetic structure among the FMDV serotype A sequences, with the African isolates resolving into established genotypes. Although the detection of serotype A is inconsistent with earlier reports, the phylogenetic clustering of the study isolates into a distinct lineage separate from previously characterized Nigerian strains suggests ongoing viral evolution and diversification within the A/Africa genotype IV topotype. This finding highlights the dynamic nature of FMDV transmission in endemic settings and underscores the necessity for continuous molecular surveillance. This pattern mirrors prior findings, where novel FMDV strains clustered separately from the earlier reported isolates within the same lineage [19, 59]. Within the study cluster, sequences from the same states group tightly together, with short branch lengths, supporting local transmission within states. The clustering of sequences from Plateau, Sokoto, Nasarawa, and Niger States indicates that the outbreaks were likely driven by pastoralist animal movement and sustained through linked herds, shared grazing areas, and local livestock trade, especially during the Muslim festive period [9, 10, 27, 67]. The observed divergence from the G-IV lineage demonstrates ongoing viral evolution. This highlights the dynamic nature of FMDV circulation in endemic areas and underscores the need for continuous molecular surveillance to capture emerging variants that may have implications for vaccine design and control strategies.

FMDV studies in Nigeria have indicated through serological assay that other serotypes (SAT 3 and Asia 1) are circulating in Nigeria (Ehizibolo et al. [11]; [56]). In this present study, our molecular analysis identified PX872161 (A/NGA/Plateau/42), PX872163 (A/NGA/Plateau/27), PX872165 (A/NGA/Sokoto/S64), and PX872166 (A/NGA/Niger) as FMDV serotype A. Although they gave Asia 1 during genotyping, BLAST analysis of the sequences generated revealed that the four sequences belonged to serotype A, sharing approximately 93% nucleotide identity with a serotype A strain (accession number MT211641) reported in 2017. To further exclude any form of ambiguity in our result, the same samples were re-tested using serotype A-specific primers, and VP1 sequencing again confirmed their classification as serotype A. Notably, the four sequences generated with both primer sets showed 100% nucleotide homology. Given the known propensity of FMDV for interserotypic recombination [5, 24, 34], the possibility of a recombinant virus was also investigated. Recombination analysis detected no evidence of recombination between our dataset (serotype A) and the Asia 1 sequences included in the analysis. However, evidence of recombination was detected within our study sequences, representing an unexpected but noteworthy finding of this study. The discrepancy between initial molecular serotyping and subsequent sequence-based confirmation, where samples initially recorded in this study as serotypes A, C, and Asia 1 were ultimately confirmed as serotype A. Given the high mutation rate and continuous evolution of FMDV, primer-template mismatches and reduced primer specificity may occur, particularly when serotype-specific assays are applied to contemporary field isolates. Although serotype-specific RT-PCR remains useful for rapid preliminary screening [36], our findings highlight its limitations in accurately discriminating among co-circulating FMDV serotypes. This inconsistency reflects limitations in serotype-specific assays with cross-reactivity potential or the presence of antigenic variants with mutations affecting primer or probe binding regions. This consequently have strong implications for FMDV surveillance. Furthermore, the results emphasize the importance of sequence-based confirmation for reliable serotype identification, molecular epidemiology, and vaccine-matching studies, particularly in regions with high viral diversity. Inaccurate serotyping may compromise outbreak surveillance and disease control strategies, emphasizing the need for continuous evaluation and optimization of diagnostic primers against currently circulating FMDV strains.

Recombination is recognized as a major evolutionary mechanism driving the genetic diversity of FMDV and has been widely reported across a broad range of FMDV genomes [49]. In this study, the finding suggests that co-circulation of genetically distinct strains may facilitate recombination events, potentially leading to the emergence of variants with altered fitness, transmissibility, or antigenic properties. Such evolutionary processes may complicate disease control efforts and warrant further investigation.

In the present study, we identified evidence of an intraserotypic recombination within the VP1 coding region of a serotype A strain (A/NGA/Plateau/27). This finding suggests that co-circulation of genetically distinct strains may facilitate recombination events, potentially leading to the emergence of variants with altered fitness, transmissibility, or antigenic properties with capacity to evade host immune responses induced by one or both parental viruses (Tosh et al. [58]). Notably, the two putative parental viruses involved in this recombination originated from Sokoto and Niger states. This spatial separation suggests that the recombination likely occurred following interstate animal movement, possibly driven by nomadic pastoral practices and livestock trade, which are known risk factors for FMDV spread in Africa [9, 27, 41, 57]. Although recombination in the FMDV genome has been reported to occur more frequently in non-structural genes and less commonly in capsid-encoding regions (Jackson et al., [23]; [7, 48, 49],), capsid recombination events have been documented and may have important implications for virus fitness and antigenic variation (Tosh et al. [58], [20],). The recombination is thought to be promoted by RNA polymerase slippage in regions with high sequence similarity. Furthermore, interserotypic recombination between serotype A and Asia 1 have been reported in some FMDV studies from Asia [24], highlighting that such events, while uncommon, are biologically plausible and epidemiologically relevant. The findings in this study therefore provides evidence for the circulation of a recombinant FMDV serotype A in Nigeria despite the suggestion by previous studies that the incursion of new FMDV strains in the country may be linked to livestock movement and cross-border trade with neighbouring countries [13, 59]. Such evolutionary processes may complicate disease control efforts and warrant further investigation.

The limited number of amino acid substitutions observed across the VP1 neutralizing antigenic regions suggests strong structural and functional conservation among the study sequences. Although non-conservative changes were detected in the B-C loop of PX872163 (A/NGA/Plateau/27), these were restricted to a double substitution, indicating limited diversification in this region. As expected, we observed greater diversity within the G-H loop, which is known to be the most antigenically flexible region of VP1 [39, 44]. The shared substitutions among the sequences point to evolutionary pressures acting on the circulating strains. In contrast, the C-terminal region remained largely conserved, with only few changes, supporting its functional stability. Despite a positional shift of the RGD motif to residues 138–140 compared with other FMDV studies [19, 49] describing positions 144–146, complete conservation of the motif across all the study sequences suggests that integrin-binding capacity is preserved. However, the two leucine (L) residues located at positions + 1 and + 4 downstream of the RGD motif, which play a key role in cell recognition, showed conservative substitution, with the first leucine residue being replaced with methionine (M). Similar mutations within this motif have been reported in some serotypes including serotype A strains [24, 49, 68].

Evidence of episodic positive selection on the VP1 protein coding gene was detected in our analyses, with the selected sites coinciding with the three major neutralizing antigenic regions: the B-C loop, G-H loop, and C-terminus. We identified two codon sites (40 and 54) within the B-C loop, four sites (132, 136, 143, and 160) within the G-H loop, and two sites (197 and 200) within the C-terminal region. The remaining positively selected sites were located outside recognized functional domains and showed moderate conservation. The major neutralizing antigenic sites are critical, as they mediate attachment to host cell receptors and facilitate viral entry into susceptible cells [70]. The clustering of the positively selected sites within the loop regions of the VP1 is in agreement with previous studies that used monoclonal antibody escape mutants and serum immunoglobulin fractionation to identify FMDV antigenic epitopes [21, 39]. Given that the statistical power of the tools used for this analysis are not fully defined, it is unlikely that all codon sites subject to positive selection were detected. The capacity of the tools to identify positively selected sites depends on several factors, including the number and relative divergence of the sequences analyzed, the strength of the phylogenetic signal, and the suitability of the nucleotide substitution model applied [21, 32]. SLAC is generally regarded as highly conservative method and may fail to detect positively selected sites in datasets characterized by limited sequence divergence or small sample sizes (Kosakovsky & Frost 2005) [32]. In contrast, FEL provides greater sensitivity for detecting pervasive selection at individual codon sites but assumes that selection pressure acts consistently across all phylogenetic lineages (Kosakovsky & Frost 2005) [32]. MEME, however, is specifically designed to detect episodic diversifying selection occurring along only a subset of branches within a phylogeny, thereby increasing its sensitivity for identifying transient or lineage-specific adaptive events [42]. This methodological distinction may explain the substantially higher number of positively selected sites identified by MEME in the present study. Taken together, these findings suggest that episodic selection may contribute significantly to the evolution of the analyzed VP1 sequences. Since most of the positively selected sites identified in this study are localized within neutralizing antigenic regions, Substitutions at these sites may facilitate immune escape and contribute to vaccine mismatch, thereby promoting adaptive evolution of the virus [37, 42, 49]. However, studies with similar results have suggested that emerging antigenic mutants may acquire a selective advantage through improved interaction with the host immune system, either during active infection or when transmitted to animals with prior immune exposure [21].

Immunoinformatics analysis of the serotype A isolates identified in this study revealed five putative B-cell epitopes within the VP1 capsid protein. Earlier studies have suggested that the major antigenic determinants of the FMDV VP1 protein are located within the G–H loop [2, 22, 45]. Consistent with these reports, our analysis also identified the epitope _124_(YNGTNKYSVSASPRRGDMGALAARIA)_149_ within the G–H loop. Three epitopes—_1_(SADPVTATVENYGGETQVQRRHH)_23_, _185_(LPVEVTTDRHKQKIIAPAK)_203_, and _162_(RAESIQE)_168_—showed few mutation combinations and are located in the N-terminus, C-terminus, and in a region adjacent to the G–H loop. These regions may represent promising targets for the development of multi-epitope-based vaccines (MEBVs) [40, 61]. Such vaccine strategies incorporate selected immunogenic epitopes capable of stimulating both humoral and cell-mediated immune responses, potentially providing broader protection against diverse circulating FMDV strains [50]. Overall, our results are consistent with previous studies that characterized similar epitopes within the VP1 protein of FMDV serotype Asia 1 [2, 71]. However, the present study additionally identified a novel epitope, _162_(RAESIQE)_168_, located adjacent to the G–H loop region of the VP1 capsid protein. This epitope may indicate ongoing antigenic evolution and adaptive changes among the circulating strains, potentially enabling the virus to maintain antigenic flexibility under host immune pressure.

## Limitation

Due to the gap in time between detection and serotyping, long storage of the samples, and inconsistent power supply, we could only proceed to serotyping with 44 samples, and we could not recover any of the positive samples from Adamawa and Oyo states. However, our sequences were from samples collected from four states located in two geopolitical zones of Nigeria. Also, all the analyses performed in this study were restricted to VP1 gene sequences. Whole-genome sequencing would likely provide a more comprehensive understanding of the virus’s phylogeography and recombination patterns. Furthermore, the predicted B-cell epitopes identified in this study were characterized using in silico analyses; future studies should validate these findings using experimental approaches, including wet-lab epitope validation, vaccine cross-neutralization, and longitudinal surveillance to assess epitope conservation over time.

### Conclusion and future directions

While previous studies in Nigeria have reported the circulation of FMDV serotype A belonging to the Africa G-IV lineage, this study provides the first evidence of a recombinant FMDV strain and identifies novel VP1 epitopes, highlighting ongoing viral evolution and potential implications for vaccine design. In the absence of a consistent and effective vaccination strategy, viral populations are primarily shaped by naturally acquired immunity, creating conditions that favour antigenic variation and genetic exchange. To better understand these dynamics, future studies should prioritize whole-genome sequencing and explicitly incorporate recombination analysis. Such approaches will provide a complete picture of FMDV evolution and clarify the extent and impact of recombination among the serotypes circulating in Nigeria.

## Data Availability

No datasets were generated or analysed during the current study.

## Declarations

### Declaration of Helsinki

This study adhered to the Declaration of Helsinki as consent was obtained from all farmers before using their animals.
